# Supplementary material for: Dissipative Particle Dynamics Simulation for the Self-Assembly of Symmetric Pentablock Terpolymers Melts under 1D Confinements
Source: Polymers (Basel). 2023 Oct 3;15(19):3982. doi: 10.3390/polym15193982 (PMC10575399; doi:10.3390/polym15193982)
Supplement: Supplementary file 1 [file polymers-15-03982-s001.zip › polymers-2584995-supplementary.pdf]

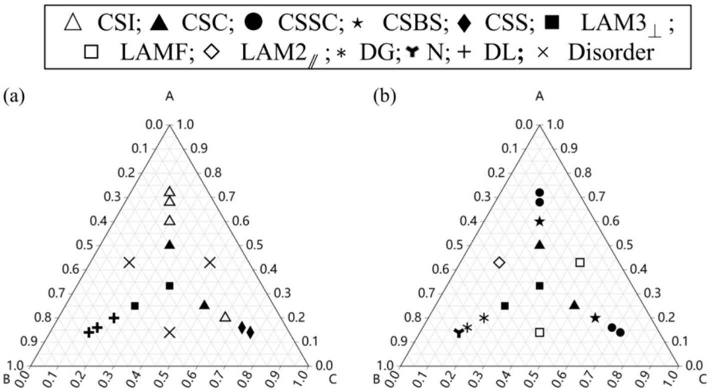

Figure S1. Phase triangle of CBABC linear pentablock terpolymers in terms of three compositions f_A , f_B , and f_C at different polymer-polymer and polymer-wall interaction strength. (a) $a_{AB} = a_{BC} = a_{AC} = 40$; (b) $a_{AB} = a_{BC} = a_{AC} = 80$.

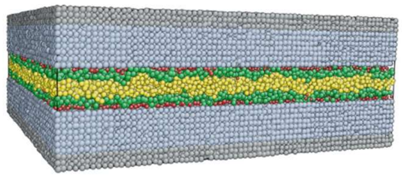

Figure S2. Parallel lamellae structure formed at $f_C = 0.14$, $f_A = f_B = 0.43$, $a_{PW} = 120$.

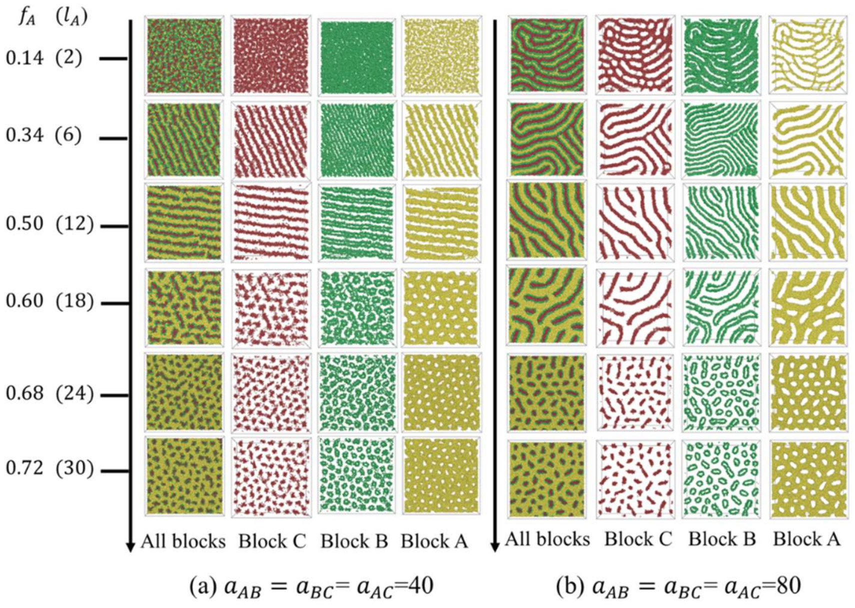

Figure S3. Phase transition sequence with f_A along $f_B = f_C$ at different polymer-polymer interaction energy(a) $a_{AB} = a_{BC} = a_{AC} = 40$; (b) $a_{AB} = a_{BC} = a_{AC} = 40$. The morphology is shown in xy plane.

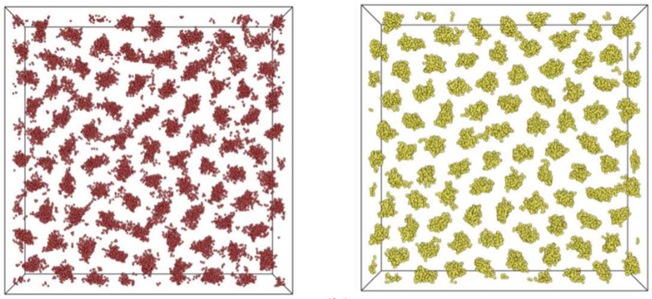

(a) (b)

Figure S4. C-cores and A-cores formed at grin point (0.68,0.16,0.16).

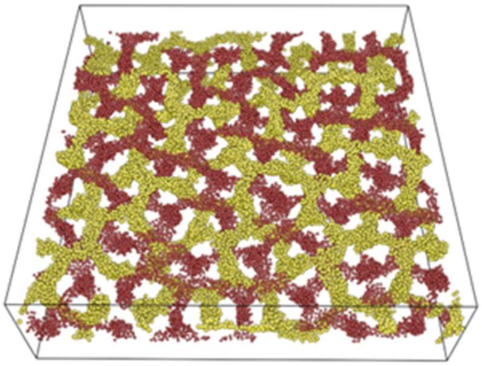

Figure S5. Double gyroid structure formed at large f_B along$f_A = f_C$ under strong polymer-polymer segregation.

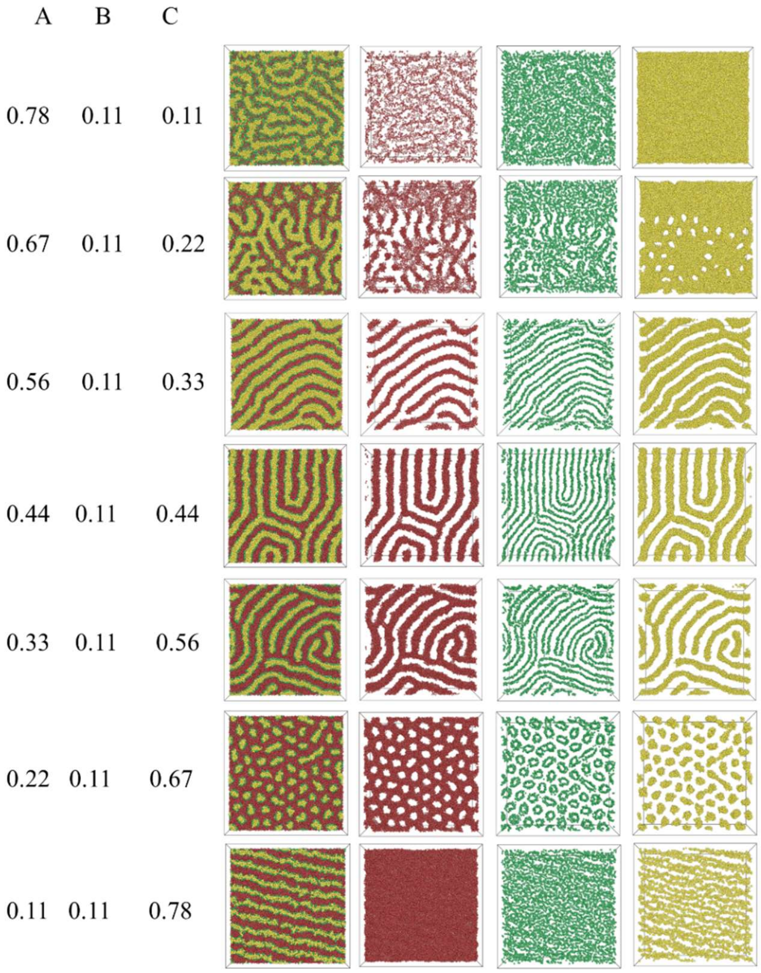

Figure S6. Phase transition sequence at $f_B = 0.11$. The morphology is shown in xy plane. The red, green and yellow colors represent C, B and A, respectively.

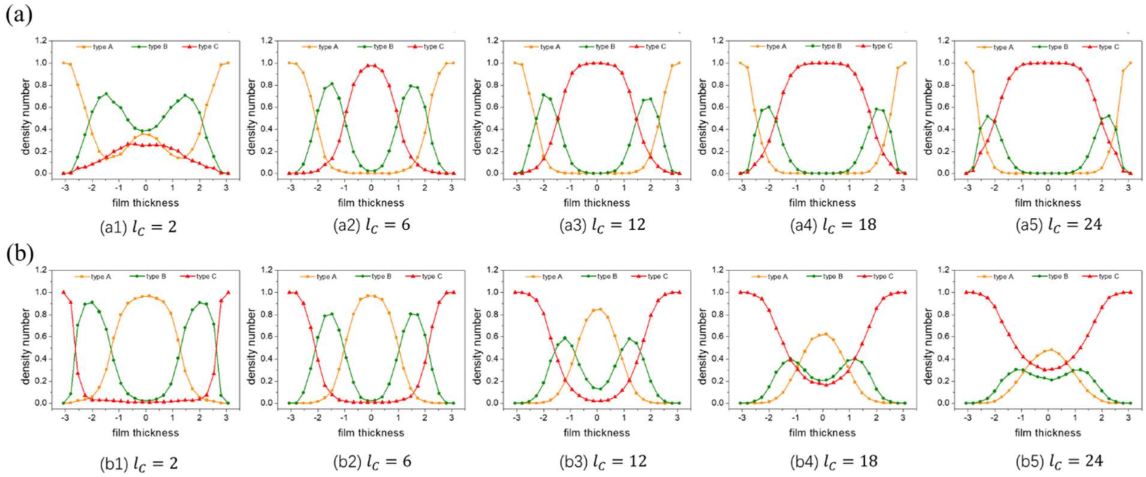

Figure S7. Density number profile for (a) A-selective wall; (b) C-selective wall as a function of C-block length l_C.

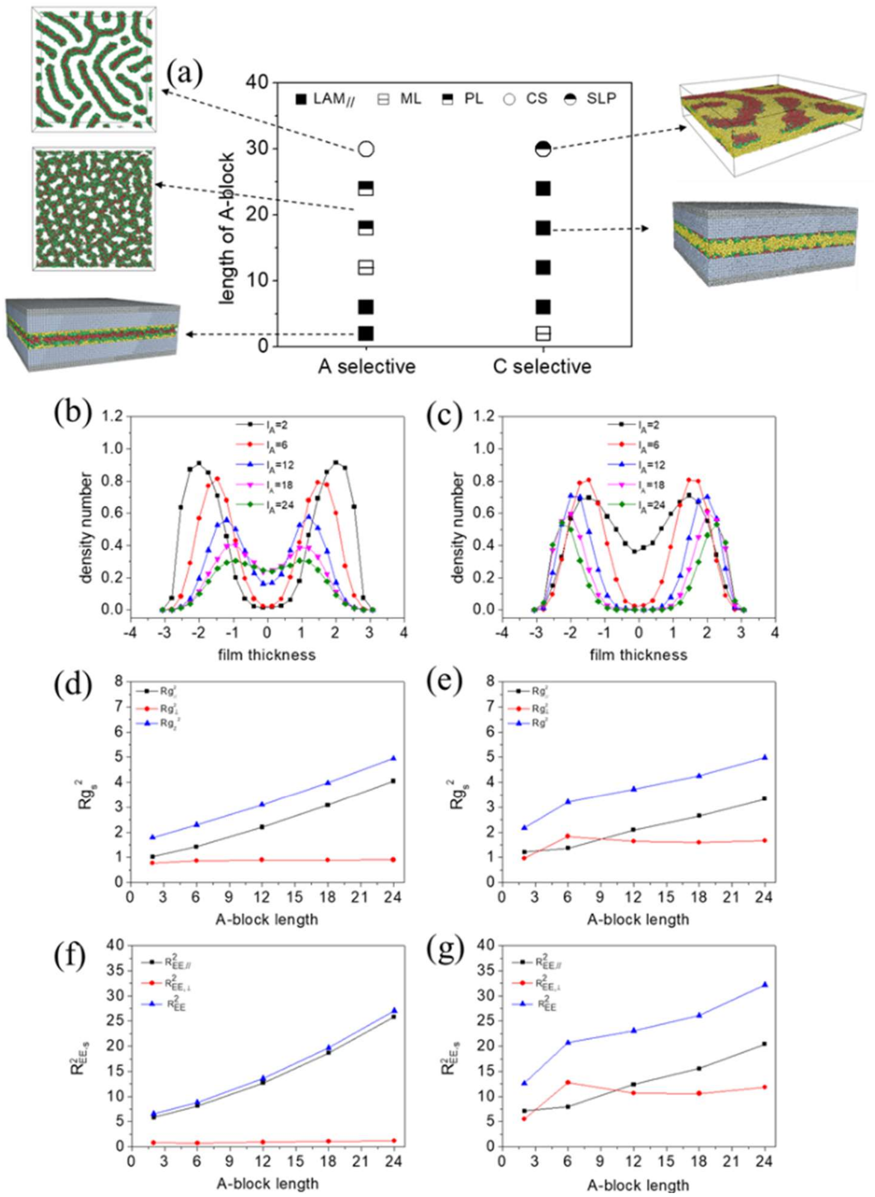

Figure S8. (a) Phase diagram of CBABC pentablock terpolymers as a function of A-block length l_A and surface preference. (b-c) Density number distribution profile for B-blocks at different lengthl_A. (d-e) Mean square radius of gyration R_g^2 as a function of block length. $R_{g,\parallel}^2$and $R_{g,\perp}^2$ are two components of R_g^2 parallel and perpendicular to the wall. (f-g) Mean square end-to-end distance R_{EE}^2 as a function of block length. $R_{EE,\parallel}^2$ and $R_{EE,\perp}^2$ are two components of R_{EE}^2 parallel and perpendicular to the wall.

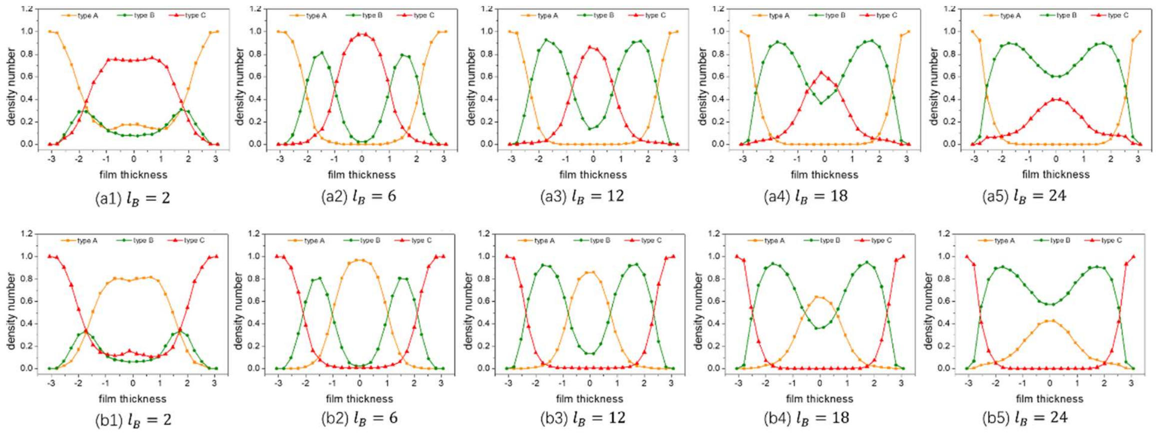

Figure S9. Density number profile for (a) A-selective wall; (b) C-selective wall as a function of B-block length l_C.
